# Long-Term Trends of Nutrients and Apparent Oxygen Utilization South of the Polar Front in Southern Ocean Intermediate Water from 1965 to 2008

**DOI:** 10.1371/journal.pone.0071766

**Published:** 2013-08-21

**Authors:** Takahiro Iida, Tsuneo Odate, Mitsuo Fukuchi

**Affiliations:** 1 National Institute of Polar Research, Midori-cho, Tachikawa, Tokyo, Japan; 2 Department of Polar Science, School of Multidisciplinary Sciences, Graduate University for Advanced Studies, Kanagawa, Japan; National Oceanic and Atmospheric Administration/National Marine Fisheries Service/Southwest Fisheries Science Center, United States of America

## Abstract

The variation of nutrients over decadal timescales south of the polar front in the Southern Ocean is poorly known because of a lack of continuous observational data in this area. We examined data from long-term continuous hydrographic monitoring of 43 years (1965–2008) in the Indian sector of the Southern Ocean, via the resupply of Antarctic stations under the Japanese Antarctic Research Expedition and Australian Antarctic Research Expedition. We found significant increasing trends in phosphate and nitrate, and a decreasing trend in apparent oxygen utilization (AOU) in intermediate water (neutral density = 27.8–28.1 kgm^−3^) south of the polar front. The rates of phosphate and nitrate increase are 0.004 µmol yr^−1^ and 0.02 µmol yr^−1^, respectively. The rate of decline of AOU was 0.32 µmol yr^−1^. One reason for this phosphate and nitrate increase and AOU decline is reduced horizontal advection of North Atlantic Deep Water, which is characterized by low nutrients and high AOU. The relationship between climate change and nutrient variability remains obscure, emphasizing the importance of long-term monitoring.

## Introduction

The Southern Ocean is very important in global ocean circulation and climate change. Decadal changes in temperature and salinity have recently been reported. Levitus [1] conveyed changes in heat content, with an increase of ∼20×10^22^ J between the surface and 3000 m, from the mid-1950s to the mid-1990s. The some study mentioned that the observed warming was likely to be a combination of natural variability and anthropogenic effects, and noted the sparsity of data coverage across the Southern Ocean. A detailed report was published by Gille [2], in which they asserted greater warming at mid depths in the Southern Ocean based on comparison of data from autonomous floats drifting between 700 m and 1000 m and earlier ocean station data. Furthermore, a long-term observational studies reported decreasing salinity in the intermediate water on the decadal scales [3], and warming of intermediate water has been reported [4]. Water mass changes have been detected in the southern Indian Ocean [5]. The Southern Ocean south of the Polar Front area is also very important, as one of the origin points of the thermohaline circulation. Polynyas are also important in the Southern Ocean for deep and intermediate water [6], [7]. Tamura et al. [8] reported that ice production where decreased by 30% from the 1990s to the 2000s, this represents one candidate for causing the recent freshening of Antarctic Bottom Water (AABW) [9], [10], which also affects deep ocean nutrients and carbon cycles in the Southern Ocean. Warming trends have also been found in the Weddell Sea in long-term monitoring datasets because of these deep water mass changes [11].

Effects of environmental changes on the interannual/spatial variation of marine biology in the Southern Ocean have also been reported [12], [13], [14], [15], [16], [17]. Hirawake et al. [13] suggested that sea surface chlorophyll *a* (chl *a*) concentrations have a 4–7 year cycles of interannual variability in the Indian sector of the Southern Ocean. Lovenduski and Gruber [18] revealed strong westerly winds over the Polar Frontal zone and Antarctic Divergence zone, associated with positive phases of the Southern Annual Mode (SAM). These strong winds drive increased northward Ekman transport of nutrient-rich deep water, which can affect the phytoplankton distribution as far north as the Polar Front [19]. However, mechanisms of driving these biological changes remain unclear. Jacobs [20] summarized the recent status of the Southern Ocean, but this applied to the area north of the Polar Front area only, and very sparse and irregular. Trends south of the Polar Front area are unknown.

Interaction between the atmosphere and ocean is very important for long-term variability of marine environments. Previous studies have described the dominant interannual climate variations in the Southern Ocean. One is the Antarctic Circumpolar Wave (ACW) [21], and the other is the Antarctic Dipole (ADP) and associated Antarctic Oscillation (AAO) and SAM [22], [23]. The former is an eastward propagation of sea ice distribution, sea surface temperature (SST) and sea level pressure (SLP) with 4-year cycle around the Southern Ocean. However, a recent meteorological study showed that the ACW was only evident from 1984 to 1994. In other periods, the AAO and Pacific-South American (PSA) teleconnection pattern has been the dominant driver of interannual variability of sea ice concentration in the Southern Ocean [24]. It is known that the PSA pattern is influenced by El Niño Southern Oscillation (ENSO) events [25], so the ENSO should also affect the marine environment in the Indian Ocean sector of the Southern Ocean. In addition, the SAM is an important mode of climate variability in the Southern Ocean on interannual timescales [18]. SAM is associated with north-south movements and intensity of the westerly winds. SAM index variability affects changes in ocean circulation [26], sea ice [27], and CO_2_ uptake and primary productivity [18], [27], [28]. From 65–70°S, sea ice concentration together with the SAM explains 51% of the variance in chl a [29].

The aim of this study is to describe long-term variability of nutrients and oxygen south of the polar frontal area in the Southern Ocean, based on long-term monitoring by the Japanese Antarctic Research Expedition (JARE) and Australian Antarctic Research Expedition (ANARE) in the Indian Ocean sector. In this paper, we analyse long-term macronutrient concentrations and dissolved oxygen data from JARE and ANARE cruises between 1965 and 2008, and present the decadal-scale variations in phosphate, nitrate and apparent oxygen utilization (AOU). Then, we demonstrate their relationships to climate change and variability in deep sea circulation in the Indian Ocean sector.

### Data Sources and Methods

The JARE has conducted monitoring observation since 1965 in the same season, using icebreakers Fuji (1965–1983) and Shirase (1983–2008) with a view to discovery long-term trends in the oceanic environment in the Indian Ocean sector of the Southern Ocean. All hydrographic and nutrient data have been reported on publications of the National Institute of Polar Research (NIPR) (http://ci.nii.ac.jp/vol_issue/nels/AA10457766_en.html). The ANARE has conducted marine science research cruises since the 1980s, using Nella Dan (1962–1987) and Aurora Australis (1989 to present). An international collaborative research project was initiated linking there datases to be creat a marine environment database that can be used as a benchmark for marine ecosystem change. This long-term database is used here to investigate trends in the marine environment.

**Figure 1:**
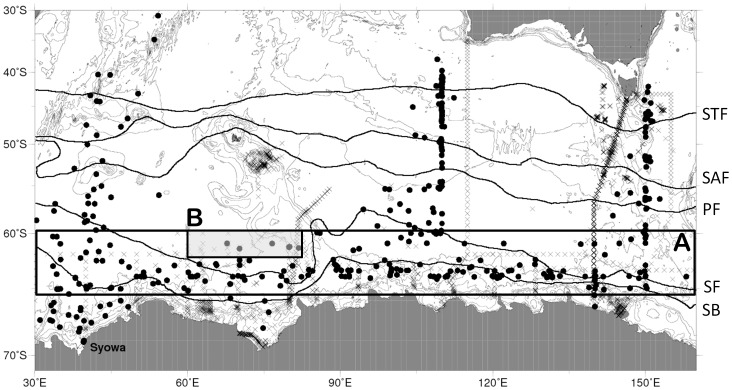
Sampling points of JARE cruise in Southern Ocean from 1965–1998. Circles indicate JARE stations and crosses ANARE stations. Overlaid are fronts in Southern Ocean, subtropical front (STF), sub-Antarctic front (STF), polar front (PF), south of Antarctic circumpolar front (SF), and southern boundary (SB).

We analyzed nutrient data sampled along cruise tracks and by stations during the period between early December and mid-February, from 1965 to 2008. All hydrographic stations from both countries are shown in Figure 1. This period was chosen to replace seasonal variability as JARE observations were made in austral summer every year, but ANARE cruises were year-round. As first step, we filtered the data using the following method to remove “bad” or “anomalous” values. Then, calculated average values for depths greater than 2000 m, under the assumption that deepwater values have been stable over decadal time scales. Average values were the same in the World Ocean Atlas (WOA09) and World Ocean Circulation Experiment (WOCE) datasets. If observed values deeper than 2000 m at each station were anomalously different (i.e., values greater than ±2*SD), we considered them to be poor quality. Second, we checked the number of poor values from each cruise, and if there were many such values from the same cruise, we excluded all data from this cruise from the analysis. Third, we constructed vertical profiles at each station, and compared differences from neighboring stations. If we found systematic errors in the profiles, we removed bad values. Fourth, we cross-checked JARE and ANARE data using the same period and area, to verify that there was no systematic error in each.

We selected as our analysis area a broad region in the Indian Ocean sector of the Southern Ocean (60°S–64°S, 30°E–160°E). This domain is located between the Polar Front and Southern Boundary (SB) Front. To reduce the effect of frontal motion, we divided the analysis area into region A (60°S–65°S, 30°E–160°E) and region B (60°S–62°S, 60°E–80°E), and compared nutrient concentrations in the two regions, following Aoki et al. [4]. Region A included 241 stations and region B 21 stations from 1965 to 2008. All datasets were interpolated to 10 m intervals, and neutral density (γ_n_) was calculated from potential temperature and salinity. All conductivity temperature and depth (CTD) salinity data were calibrated by an Autosal laboratory salinometer (Ocean Scientific International Ltd., Hampshire, UK).

Water samples were taken from 24 depths above 3000 m (including the surface), using a bucket for surface samples and Niskin bottles for the other depths. NO_3_, PO_4_ and SiO_2_ were measured by the molybdenum blue, cadmium column and molybdenum yellow reduction methods, respectively [30], [31]. Dissolved oxygen was measured by the Winkler method.

## Results

**Figure 2:**
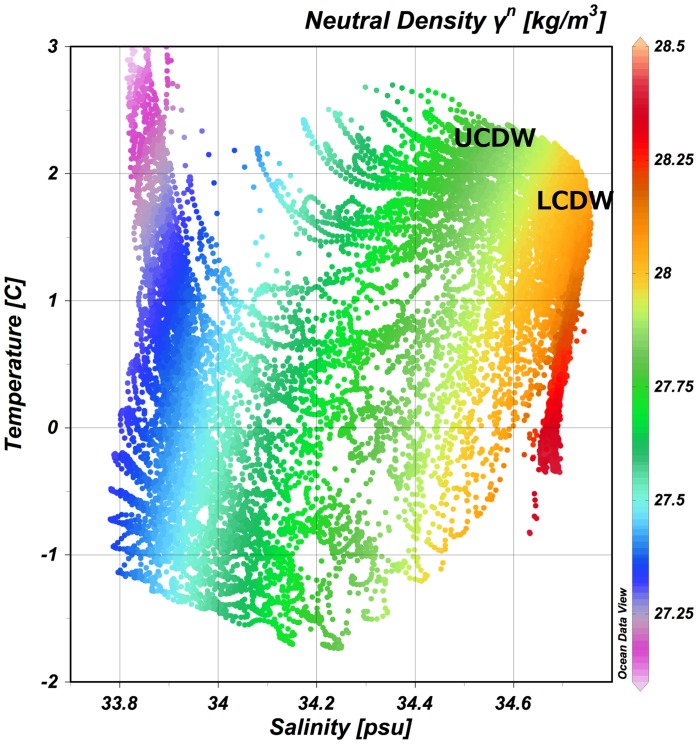
T-S-neutral density diagram for study area.

We investigated long-term phosphate, nitrate, silicate and apparent oxygen utilization (AOU) using the aforementioned datasets. First, a temperature-salinity diagram for region A is shown in Figure 2. Each plot indicates the yearly mean value of each component. Low-salinity water covered the surface layer, and a strong halocline developed in the upper 200 m of this region. Under the halocline, Intermediate Water was defined as Upper Circumpolar Deep Water (UCDW) from 200–800 m depths, which was characterized with neutral density (γ_n_ = 27.35 to 27.75) [32]. UCDW is characterized by an oxygen minimum (O_2_<4.5 ml l^−1^). Lower circumpolar deep water (LCDW) is distributed under UCDW, which was characterized by a salinity maximum and nutrient minimum and whose source is North Atlantic Deep Water (NADW) [33], [34]. UCDW was observed as a temperature maximum (>1.5°C) layer in the vertical profile [35], [36]. Our analyzed datasets were mostly from around 64°S. This area is known to be near the SB Front, and the Southern Antarctic Circumpolar Current Front (SACCF) [35], and is south of the Antarctic Divergence upwelling area.

**Figure 3:**
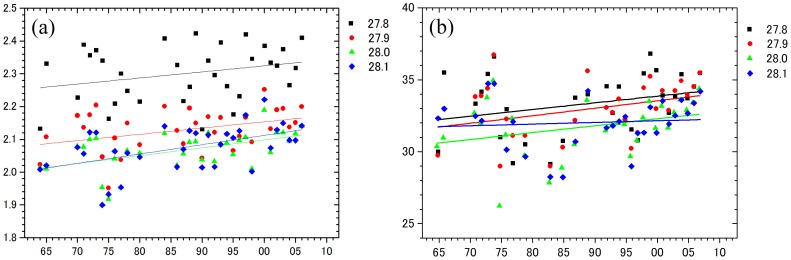
Time series of yearly averaged phosphate (a) and nitrate (b) concentrations in area A intermediate water (γ_n_ = 27.8–28.1).

**Figure 4:**
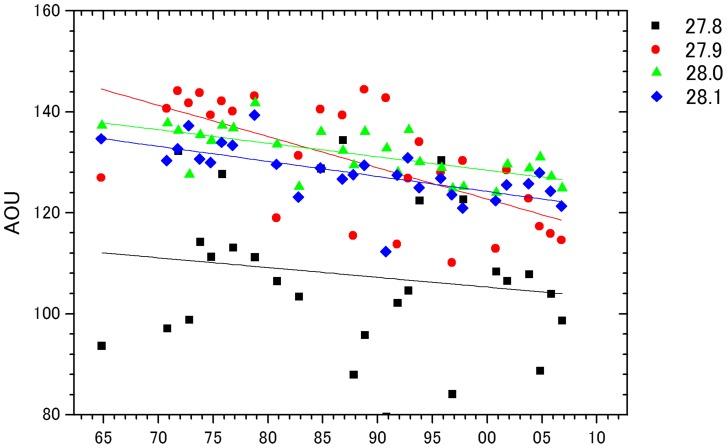
Time series of yearly averaged apparent oxygen utilization (AOU) in area A intermediate water (γ_n_ = 27.8–28.1).

Temporal variability of phosphate, nitrate and silicate concentrations are shown on each surface of neutral density γ_n_ in area A (Fig. 3). We found significant linear increases of 0.004 µmol/kg yr^−1^ PO_4_ (p<0.05) and 0.02 µmol/kg yr^−1^ NO_3_ (p<0.05) in γ_n_ = 27.8–28.1 water. Statistical parameters are summarized in Table 1, and the number of data points, mean values, maximum and minimum values of PO_4_ in each neutral density are presented (Table 2). Although the observations were relatively limited from 1960s to 1970s, sufficient measurements were recorded from the 1980s. The most significant increasing trend of nitrate and phosphate were observed in γ_n_ = 27.9–28.0 water. This water was distributed at depths 500–1000 m, which was characterized by a temperature maximum. Figure 4 shows long-term trends of AOU, which significantly declined in γ_n_ = 27.9 water. We did the same analysis for area B, revealing the same trend as area A. However, the trend was not significant because of a lack of sufficient sampling stations (21 in area B). Decadal-scale periodicity was found for nutrients and AOU (Fig. 3). There was a positive correlation between nutrients and AOU, but this was not statistically significant.

**Table 1:** Statistical parameters (slope, r^2^ and p values) of PO_4_, NO_3_ and AOU trends.

	PO_4_			NO_3_			AOU		
γ	slope	r^2^	p	slope	r^2^	p	slope	r^2^	p
27.8	0.0027	0.0965	2.99E-10	0.0898	0.1733	1.44E-08	–0.2945	0.0266	2.22E-29
27.9	0.0035	0.1515	2.85E-10	0.0707	0.0835	3.51E-12	–0.9394	0.4367	6.45E-40
28.0	0.0044	0.2908	2.19E-10	0.0768	0.1230	5.55E-12	–0.3952	0.4377	2.89E-45
28.1	0.0051	0.2653	2.54E-10	0.0263	0.0177	2.59E-11	–0.4377	0.4150	2.91E-46

**Table 2:** The number of data points, mean values, maximum and minimum values of PO_4_ in each neutral density.

	PO4(γ_n_ = 27.8)				PO4(γ_n_ = 27.9)				PO4(γ_n_ = 28.0)				PO4(γ_n_ = 28.1)			
	data point	mean	minimum	maximum	datapoint	mean	minimum	maximum	datapoint	mean	minimum	maximum	datapoint	mean	minimum	maximum
1965	5	2.13	2.06	2.2	7	2.11	2.05	2.17	13	2.10	1.98	2.25	12	2.10	1.95	2.19
1966	2	2.33	2.27	2.39	2	2.24	2.19	2.29	2	2.10	2.09	2.11	2	2.12	2.11	2.12
1967	ND	ND	ND	ND	ND	ND	ND	ND	ND	ND	ND	ND	ND	ND	ND	ND
1968	ND	ND	ND	ND	ND	ND	ND	ND	ND	ND	ND	ND	ND	ND	ND	ND
1969	ND	ND	ND	ND	ND	ND	ND	ND	ND	ND	ND	ND	ND	ND	ND	ND
1970	ND	ND	ND	ND	ND	ND	ND	ND	ND	ND	ND	ND	ND	ND	ND	ND
1971	3	2.23	2.08	2.42	6	2.33	2.31	2.37	7	2.20	2.14	2.27	2	2.20	2.14	2.25
1972	6	2.39	2.29	2.44	9	2.29	2.17	2.44	10	2.20	2.13	2.26	6	2.17	2.02	2.28
1973	5	2.36	2.22	2.48	5	2.34	2.28	2.44	10	2.23	2.16	2.32	4	2.26	2.22	2.3
1974	14	2.35	2.08	2.53	22	2.38	2.22	2.5	28	2.23	2.08	2.34	16	2.26	2.14	2.38
1975	1	2.34	2.34	2.34	4	2.15	2.09	2.26	4	2.02	1.97	2.11	3	1.94	1.93	1.95
1976	9	2.16	1.92	2.34	9	2.02	1.77	2.26	13	1.98	1.8	2.14	11	1.99	1.7	2.16
1977	6	2.21	1.86	2.39	8	2.24	2.17	2.31	12	2.14	1.96	2.29	8	2.18	2.08	2.25
1978	3	2.30	2.19	2.4	2	2.14	2.13	2.15	2	2.02	1.98	2.06	1	2.02	2.02	2.02
1979	12	2.25	2.09	2.42	23	2.30	2.12	2.42	27	2.18	1.92	2.32	18	2.17	2.09	2.28
1980	ND	ND	ND	ND	ND	ND	ND	ND	ND	ND	ND	ND	ND	ND	ND	ND
1981	4	2.22	2.02	2.37	7	2.21	2.04	2.26	13	2.17	2.04	2.31	9	2.15	2.07	2.24
1982	ND	ND	ND	ND	ND	ND	ND	ND	ND	ND	ND	ND	ND	ND	ND	ND
1983	6	2.43	2.14	2.64	13	2.48	2.34	2.64	16	2.42	2.27	2.57	11	2.42	2.23	2.68
1984	ND	ND	ND	ND	ND	ND	ND	ND	ND	ND	ND	ND	ND	ND	ND	ND
1985	4	2.41	2.34	2.45	10	2.37	2.2	2.5	11	2.25	2.02	2.34	5	2.29	2.25	2.39
1986	ND	ND	ND	ND	ND	ND	ND	ND	ND	ND	ND	ND	ND	ND	ND	ND
1987	8	2.33	2.05	2.49	16	2.27	2.12	2.41	16	2.11	2.02	2.2	11	2.11	2.02	2.25
1988	18	2.22	1.95	2.6	34	2.21	2	2.56	53	2.17	1.89	2.39	46	2.18	1.96	2.33
1989	4	2.32	2.19	2.41	8	2.37	2.24	2.46	15	2.22	2.13	2.35	7	2.27	2.16	2.32
1990	29	2.42	2.3	2.55	32	2.29	2.17	2.48	34	2.23	2.12	2.38	22	2.26	2.12	2.4
1991	3	2.13	2.01	2.32	4	2.15	2.06	2.26	8	2.14	1.91	2.27	3	2.11	1.84	2.26
1992	16	2.34	2.17	2.5	45	2.33	2.12	2.48	48	2.26	2.02	2.46	38	2.25	2.06	2.38
1993	9	2.30	2.12	2.43	17	2.26	2.13	2.4	33	2.13	1.82	2.42	25	2.11	1.9	2.3
1994	16	2.40	2.21	2.53	29	2.32	2.2	2.49	51	2.21	2.1	2.28	39	2.21	2.14	2.28
1995	22	2.26	2.04	2.48	31	2.25	2.09	2.44	39	2.23	2.12	2.32	40	2.25	2.16	2.31
1996	21	2.42	2.13	2.53	42	2.32	2.13	2.51	61	2.21	2.15	2.31	42	2.24	2.15	2.29
1997	9	2.23	2.11	2.44	16	2.24	2.12	2.37	22	2.22	2.13	2.28	23	2.26	2.18	2.31
1998	30	2.42	2.11	2.91	43	2.33	2.08	2.5	72	2.22	2.07	2.55	49	2.33	2.04	3.16
1999	11	2.38	2.1	2.5	17	2.22	1.9	2.4	17	2.10	1.8	2.3	9	2.09	1.8	2.3
2000	3	2.63	2.57	2.66	3	2.45	2.4	2.5	3	2.32	2.28	2.34	3	2.31	2.28	2.35
2001	19	2.48	2.28	2.69	28	2.45	2.27	2.69	46	2.36	2.2	2.57	22	2.40	2.26	2.59
2002	16	2.33	2.12	2.47	25	2.28	2.12	2.44	40	2.17	2.06	2.31	25	2.19	2.07	2.28
2003	19	2.32	2.1	2.54	28	2.36	2.16	2.51	56	2.26	2.13	2.42	41	2.27	2.19	2.41
2004	33	2.37	2.17	2.68	47	2.36	2.13	2.62	71	2.26	1.92	2.42	53	2.30	2.15	2.45
2005	23	2.27	2.12	2.51	27	2.28	2.134	2.46	47	2.22	2.07	2.34	55	2.23	2.1	2.32
2006	23	2.32	2.105	2.544	41	2.30	2.146	2.73	59	2.25	2.121	2.851	49	2.22	2.142	2.335
2007	23	2.39	2.228	2.582	33	2.38	2.234	2.535	51	2.29	2.129	2.525	53	2.29	1.871	2.773

ND means no data.

## Discussion and Conclusions

We found long-term variability in phosphate, nitrate and AOU south of the polar frontal area in the JARE and ANARE historical datasets. Principal findings were as follows.

1. Increases in phosphate and nitrate in the intermediate water. Most significant increases were for water with γ_n_ = 28.0. No trends were observed for silicate.
2. A decline of AOU in the intermediate water. Most significant decreases were for water with γ_n_ = 27.9.

We considered three possible mechanisms for the observed trends in the intermediate water of the Indian sector of the Southern Ocean: (1) Variations in biological productivity in the surface layer; (2) variation in the magnitude of horizontal advection in upper layers, and (3) variations of mixing of surface and intermediate waters south of the Polar Front.

First, we consider surface biological productivity. Hirawake et al. [14] reported chl a variations from 1965 to 2002 in the same area, using JARE data. They indicated variations of 3–7 year periodic cycles and increasing trends of chlorophyll a concentrations, but the cause of such variability was obscure. Smith and Comiso [37] stated that annual productivity across the entire Southern Ocean, calculated by the satellite- based Vertically Generalized Production Model (VGPM) of Behrenfeld and Falkowski [38], has increased significantly since 1998, particularly in January and February. If surface productivity was increasing in the region, then sinking organic particles should have been increasing. As a result, decomposition by microbes should be increasing, resuting in increased nutrients and AOU. This contrasts with our results, so there is uncertainty regarding the possibility of a productivity increase.

Second, we consider variations in horizontal advection of water masses across the study region. The water with γ_n_ = 28.0 is known to have NADW origin. Low phosphate and silicate concentrations were observed in South Atlantic Deep Water (SADW) (2500 m depth), and there was a maximum AOU in this layer from the World Ocean Atlas dataset [39], [40]. However, the silicate minimum was not observed in SADW. There are significant differences between vertical nitrate and silicic acid distributions in the Southern Ocean. The nitrate gradient is vertically well mixed except for reduced concentrations within the upper 100 m or so, whereas the silicic acid gradient penetrates to 1000 m and deeper. Therefore, a decline in NADW horizontal advection would induce the phosphate and nitrate increase and AOU decline. Bryden et al. [41] reported that the Atlantic meridional overturning circulation slowed by about 30% between 1957 and 2004, whereas northward transport in the Gulf Stream across 25°N has remained nearly constant. The slowing is evident as both a 50% increase in southward-moving mid-ocean recirculation of thermocline waters, and a 50% decrease in southward transport of lower NADW between 3,000 and 5,000 m depths. This decline in southward NADW in the Atlantic Ocean could have induced the increase of phosphate and nitrate and decrease of AOU in the Southern Ocean intermediate water. However, we cannot explain the lack of a concomitant significant trend in silicate concentration. The silicate cycle is very different compared with other macro nutrient in the Southern Ocean [42]. The nutrient concentrations are high in the southern region of Southern Ocean, and decrease in the northern part. However, the Nitrate:Silicate ratio is very different in each region. Only silicate is depleted to the north of 50°S [40]. The following thought to be responsible. The deep water nutrient concentrations is high due to re-mineralization. This high nutrient water upwelled in Antarctic coastal areas of the Southern Ocean [43], where diatoms most successful grow in this area. In this case, all macro nutrients are decreasing, and organic matter sinks into deep layers. Organic matter in the deep layer is rematerialized at depths shallower than where dissolution of opal occurs due to difference of the remineralization ratio. Opal is slower to degregate than nitrate and phosphate [44]. This re-mineralized water is advected to the northern part of the Southern Ocean and, as a result, the ratio of N:Si and P:Si are different between north and south of the Southern Ocean. However, horizontal advection of NADW is very slow, and if changes of NADW are observed in the Atlantic Ocean, their effects on the Southern Ocean would not be observed for a long time. We believe that the variations iv NADW possible explanation for the Southern Ocean nutrient changes observed, but doubts remain.

Third, we considered mixing rate variability in the region. What was responsible for the change of mixing rate between surface and intermediate waters in the Southern Ocean? Deeper water contains low AOU and low phosphate compared with γ_n_ = 28.0 water. This indicates that fresher water originating from Antarctic Bottom Water (AABW) was included. Hall and Visbeck [26] suggested that to satisfy mass continuity, increased upwelling occurs around the Antarctic coast, while a 15% decrease in upwelling takes place around 45°S. The cause of this increase is thought to be a positive SAM, which induced strong westerly winds and strength of the Antarctic Circumpolar Current (ACC) [45]. As a result, northward Ekman pumping intensified, and there was increased upwelling in the Antarctic Divergence area. This induced deep high phosphate and nitrate water upwelling in the surface layer. However, if the deep water upwelling intensifies, dissolved oxygen (DO) decreases in the Intermediate Water. Aoki [46] suggested that a DO anomaly between the 1970s and 1980s indicated a decline of DO around 150°E. A modeling study indicated that the upwelling increase accelerated the DO decline in the Antarctic Divergence area [47], a result inconsistent with ours. They stated that the cause of these variations depends on ACC strength. However, negative DO anomalies were found only at 150°E; the other region (e.g.110°E) had positive DO anomalies. The study indicated that the oxygen decreases were caused by increased stratification and decreased convection resulting from warming and/or freshening of surface waters. However, this phenomenon is limited to only the eastern part of the Indian Ocean sector of the Southern Ocean. Furthermore, our dataset includes the SB area, which experiences a different mechanism. If intensity of northward Ekman transport is stronger in the divergence area, then the waters are compensated from other regions. If southern surface water is drawn down to a deeper layer, the AOU might decrease in the study area. Clearly we need to clarify the mechanisms, and a regional numerical modeling study is needed.

More analysis is also needed for the water mass exchange in this region, for example via the use of a chemical tracer (CFC or others). Overall, the observation changes in nutrient and DO environments south of the Polar Front in the Indian Ocean sector of the Southern Ocean were consistent with changes in horizontal advection in NADW. However, more observations are needed to finding a large decadal oscillation or interannual variability. The continuation of exsisting observations is crucial to monitor and predict present and future changes, in addition to the use of new methodologies such as tracer experiments and modeling.
